# Ehlers-Danlos syndrome kyphoscoliotic type 2 caused by mutations in the FKBP14 gene: an analysis of five cases

**DOI:** 10.12688/f1000research.52268.1

**Published:** 2021-06-25

**Authors:** Alla Nikolaevna Semyachkina, Ekaterina Alexandrovna Nikolaeva, Nailya Mansurovna Galeeva, Alexander Vladimirovich Polyakov, Maria Andreevna Kurnikova, Vera Аlexandrovna Belova, Irina Valerievna Shulyakova, Ilya Sergeevich Dantsev, Goar Vladimirovna Dzhivanshiryan

**Affiliations:** 1 Veltischev Research and Clinical Institute for Pediatrics, Pirogov Russian National Research Medical University, Moscow, 125412, Russian Federation; 2 Research Centre for Medical Genetics, Moscow, Russian Federation; 3 Dmitry Rogachev National Medical Research Center of Pediatric Hematology, Oncology and Immunology, Moscow, Russian Federation; 4 Center for Precision Genome Editing and Genetic Technologies for Biomedicine, Pirogov Russian National Research Medical University, Moscow, 117997, Russian Federation

**Keywords:** children, rare (orphan) disorders, monogenic connective tissue disorders, clinical findings, FKBP14 gene, c.362dupC duplication

## Abstract

**Background.** This study deals with a rare (orphan) monogenic connective tissue disorder - Ehlers-Danlos syndrome kyphoscoliotic type 2 (EDSKS2). Kyphoscoliotic type 2 Ehlers-Danlos syndrome is an autosomal recessive disorder caused by mutations in the FKBP14 gene (7p14.3), which encodes the FKBP22 protein. According to the 2017 classification, this type is in group seven - collagen spatial structure and cross-linking defects. We present results of clinical examination and molecular genetic analysis for five patients with age varying from two to fifteen years.

**Methods.** Five patients were examined using clinical and laboratory methods. DNA samples used for the analysis were extracted from whole blood samples using a Wizard® Genomic DNA Purification Kit (Promega, USA) according to the manufacturer's protocol.

**Results.** The major clinical findings were kyphoscoliosis, early motor development delay, muscular weakness, hypotonia and hearing loss. Molecular genetic analysis detected a homozygous c.362dupC duplication in exon 3 of the FKBP14 gene in all five patients. This mutation is common in various countries. Differential diagnostics were carried out to exclude other Ehlers-Danlos syndrome types and myopathies.

**Conclusions.** Literature analysis and examination of five EDSKS2 patients demonstrated the involvement of major organs and systems, such as joints, spine, muscles, cardiovascular system, respiratory system, hearing, and vision, into the pathological process. Kidney mobility increases and nephroptosis seems to be secondary caused by muscular weakness. During molecular genetic analysis, to verify EDSKS2 it is recommended to initially search for the c.362dupC duplication, which appears to be common in European countries, including Russia.

## Introduction

Ehlers-Danlos syndrome (EDS) is commonly encountered by various medical specialists. It is mainly characterized by skin hyperelasticity, joint hypermobility, easy bruising, and hypertrophic scarring. The disorder is genetically heterogeneous: according to the 2017 classification, there are 13 clinical genetic types, ^1^ divided into seven groups depending on the pathogenesis. The kyphoscoliotic type belongs to the B group – disorders of collagen folding and collagen cross-linking – or type VI in the 1997 classification. Patients with kyphoscoliotic type come to the attention of pediatricians, neurologists, and orthopaedists.

EDS kyphoscoliotic type is an autosomal recessive disorder with estimated prevalence of one per 100,000 newborns. ^2^ Its main symptoms (major clinical diagnostics criteria) are severe muscle hypotonia at birth (“floppiness”), early-onset kyphoscoliosis (usually progressive), hypermobility of joints, dislocations/subluxations (especially of knee joints). ^1^ Additional (minor) diagnostic criteria include skin hyperelasticity, easy bruising, arterial ruptures/aneurisms, osteopenia/osteoporosis, bluish sclerae, umbilical/inguinal hernia, thorax deformations, marfanoid habitus, equinovarus feet deformities, myopia.

EDS kyphoscoliotic type is currently subdivided into two subtypes. EDS kyphoscoliotic type 1 (EDSKS1) is caused by mutations in the *PLOD1* gene (1p36.22), which encodes lysyl hydroxylase, a catalyst essential for the stability of the intermolecular collagen crosslinks. ^3, 4^ EDS kyphoscoliotic type 2 (EDSKS2) is caused by mutations in the *FKBP14* gene (7p14.3), which encodes the FKBP22 protein. FKBP22 is a highly conservative peptidyl-prolyl *cis-trans* isomerase (PPIase), which catalyzes collagen folding and acts as a chaperone to collagen types III, VI, X. ^5^ Thus, both genes are tied to collagen spatial structure formation.

These two subtypes are clinically similar, but slightly different. Mutations in the *PLOD1* gene lead to moderate scarring and bruisability, microcornea, scleral and ocular ruptures, and facial abnormalities (low set ears, epicanthal folds, downslanting eyes, synophrys, high-arched palate). EDSKS2 is less explored because of its recent discovery and lower frequency. Some patients show signs of hearing loss (sensorineural, conductive or mixed) that were not present at birth, as well as follicular hyperkeratosis, muscular atrophy, and bladder diverticulum.

Because this EDS type is less frequent and not fully explored, in this study we present the clinical data of five patients with EDSKS2.

## Methods

### Clinical examination

Five patients were examined using clinical and laboratory methods in the clinical genetics department of Veltischev Research and Clinical Institute for Pediatrics, Moscow, Russia. Molecular genetic analysis was carried out in the DNA diagnostics laboratory of Research Centre for Medical Genetics, Moscow, Russia.

### DNA extraction

DNA samples used for the analysis were extracted from whole blood samples using a Wizard ^®^ Genomic DNA Purification Kit (Promega, USA) according to the manufacturer's protocol.

### *FKBP14* variant detection

The sequence of the *FKBP14* gene (( Accession number NG_032173.1 (genomic), Accession number NM_017946.4 (mRNA)) was analyzed for possible mutations via direct automated Sanger sequencing. Primer sequences, МgCl _2_ concentrations and primer annealing temperatures are presented in Table 1. PCR products were sequenced using the ABI PRISM Big Dye Terminator (v 3.1) Cycle Sequencing Kit (Applied Biosystems, Foster City, CA, USA) on an ABI3130xl Genetic Analyzer (Applied Biosystems, Foster City, CA, USA).

**Table 1.** Primer sequences and PCR conditions for *FKBP14* exons.

DNA fragment	Primer sequence	Fragment length, bp	MgCl _2_ concentration, mM	Primer annealing temperature, °С (cycle)
Exon 1	F-GTCGAGGGACCTTTCGCTGC	163	4	63 (32)
	R-GCTGGCATAAGTGAGTGGATTC			
Exon 2	F-CACTTACTGGTGGGAAAATGCAC	263	4	63 (32)
	R-CTGTCTCCTAATCCAGAGAACAA			
Exon 3	F-CATATATGACAATCTTAGGAAGGCTC	240	2	65 (32)
	R-GGAGTAGGAAGAAGGAAAGGTC			
Exon 4	F-GCTCAATGTGGGTATCTTATGAATCC	690	1.6	67 (32)
	R-GCCCTCTCTTGAAAGATGAGTGC			

Sequencing results were analyzed using BLAST (Basic Local Alignment Search Tool) ( http://www.ncbi.nlm.nih.gov/blast) to compare a subject nucleotide sequence with the database. In our research we worked with search database Human genomic plus transcript (Human G+T) using blastn ( https://www.ncbi.nlm.nih.gov/Class/MLACourse/Modules/BLAST/nucleotide_blast.html) (Optimized for somewhat similar sequences).

## Case descriptions

We examined five patients with EDSKS2: three boys and two girls with age ranging from two to fifteen years ( Table 2). In four families, the marriage did not appear to be consanguineous. However, in family number (no.) 3 (girl, 11 years) both parents had distant relatives originating from the same small village, which could potentially mean kinship.

**Table 2.** The main clinical, laboratory and functional indicators in patients with kyphoscoliotic type 2 Ehlers-Danlos syndrome.

Patient number	1	2	3	4	5
Sex	M	F	F	M	M
Age of diagnosis, years	3	8	11	15	2
Intelligence Quotient	90	95	80	100	93
Physical development	Very low, harmonic, <3 percentile	Very high, harmonic; body length and mass >75-90 percentile	Disharmonic: body length 25-50 percentile, mass >97 percentile	Very low, harmonic, <3 percentile	Disharmonic: body length 50-75 percentile, mass 10-25 percentile
Kyphoscoliosis	+	+	+	+	+
Age of kyphoscoliosis onset, months	17	12	24	24	8
X-ray data	Combined thoracolumbar kyphoscoliosis of the 2nd degree. Osteoporosis. *Spina bifida sacralis dorsalis* S3-S5	*Pectus excavatum* of the 2nd degree. S–shaped thoracolumbar scoliosis of the 3d degree. (post-surgery)	Thoracolumbar dextroscoliosis of the 3d degree. Osteoporosis.	*Pectus excavatum* of the 2 ^nd^ degree. S–shaped thoracolumbar scoliosis of the 4th degree	Thoracolumbar levokyphoscoliosis of the 2nd degree. Osteoporosis.
Hypermobility Beighton score	8	8	8	8	6
Motor development impairment	+	+	+	+	+
Gowers' sign	+	+	+	+	+
Tendon reflexes decreased	+	+	+	+	+
Cardiovascular impairments	+	+	+	+	+
Congenital heart defect	Open *ductus arteriosus*	Open *ductus arteriosus*, bicuspid aortic valve	Borderline narrowed aorta – 17 mm at sinotubular junction	-	-
Electrocardiograpic abnormalities	sinus arrhythmia	-	sinus arrhythmia	sinus tachyarrhythmia	-
Extrinsic respiratory restrictions (spirometry results)	Spirometry not performed	Profound combined respiratory pulmonary function impairment	Profound combined respiratory pulmonary function impairment	Profound combined respiratory pulmonary function impairment	Spirometry not performed
Ultrasound imaging of abdominal organs and kidneys	Bilateral nephroptosis	Bilateral nephroptosis			Nephroptosis (left side)
Hearing impairment	-	-	Mild to moderate bilateral sensorineural hearing loss	Bilateral sensorineural hearing loss	Mild bilateral mixed hearing loss
Vision impairment	Mild bilateral hypermetropia	-	-	Progressive high myopia with astigmatism, chorioretinitis (right side) without signs of inflammation	Mixed bilateral astigmatism

## Ethical approval

The study was approved by the Ethics committee of the Research and Clinical Institute of Pediatrics (approval number #2, 2021) named after Yuri Veltischev of the Pirogov Russian National Research Medical University of the Ministry of Health of the Russian Federation. A written informed consent was obtained from the participants and parents of participants under the age of 18 to take part in the study. The study was done in accordance with the principles outlined in the Helsinki Declaration (1964).

## Results

**Figure 1.**
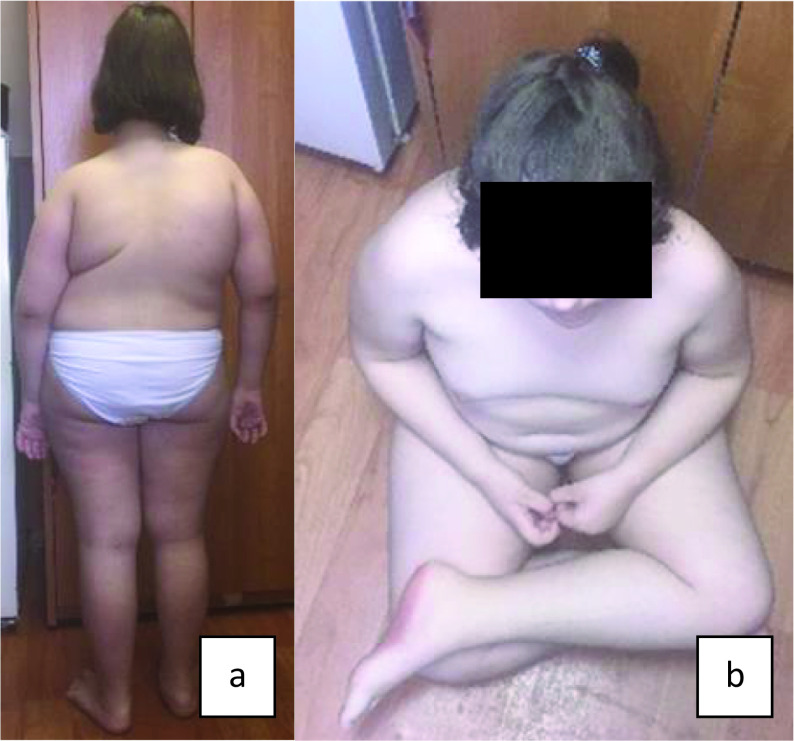
Proband no. 3: а) thoracolumbar dextroscoliosis of the 3 ^rd^ degree; b) joint hypermobility, obesity of the 1 ^st^/2 ^nd^ degree.

**Figure 2.**
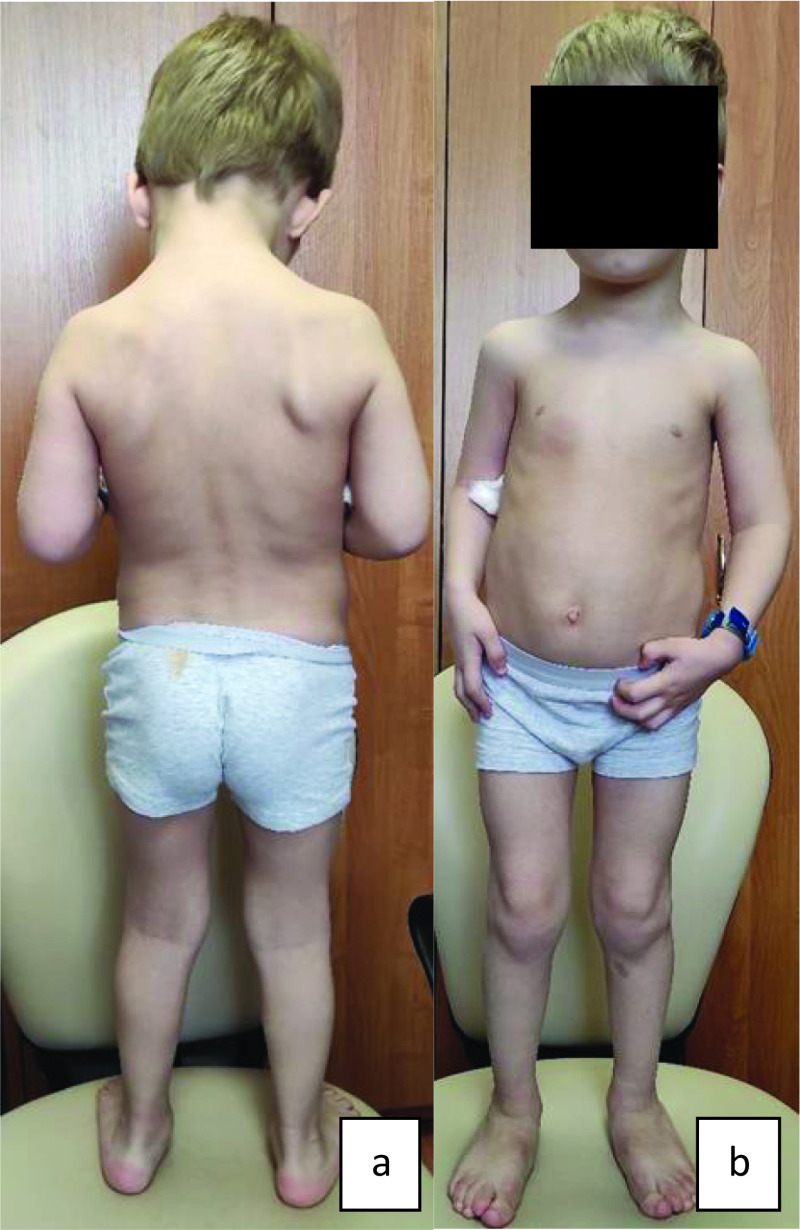
Proband no. 5: a) thoracolumbar levokyphoscoliosis of the 2 ^nd^ degree; b) epicanthic fold, *genu valgum, pes planovalgus*

All patients had moderate to severe kyphoscoliosis, early motor development delay, muscular weakness, hypotonia, and hearing impairment ( Table 2). Figure 1 and Figure 2 (a, b) show main phenotypic characteristics of patients no. 3 and 5 with EDSKS2. Physical development of the children at the time of examination varied. Probands no. 1 and 4 (three and fifteen years respectively) had very low, harmonic development (all values below third percentile). Patients no. 3 and 5 (eleven and two years respectively) had disharmonic physical development (no. 3 - average body length 25–50 percentile and very high mass >97 percentile; no. 5 - above average body length 50–75 percentile and below average mass 10–25 percentile). Patient no. 2 had high, harmonic physical development: all values above 90–97 percentile.

The age of kyphoscoliosis formation varied from eight to twenty-four months. Clinical examination and X-ray showed thoracolumbar kyphoscoliosis in all five cases. Three children (aged two, three, eight years) had kyphoscoliosis of the 2nd degree, while the older patients (11 and 15 years) had kyphoscoliosis of the 3rd and 4th degree respectively. Probands no. 2 and 4 (eight and fifteen years), aside from kyphoscoliosis, had *pectus excavatum* of the 2nd degree. Patients no. 1, 3, 4, and 5 had *pes planovalgus.* X-ray showed signs of osteoporosis in patients no. 1, 3, and 5. In addition to this, patient 1 had *spina bifida sacralis dorsalis* S3–S5.

Joint hypermobility was evaluated as eight on the Beighton scale for four patients and as six for one patient (Patient no. 5). Four patients did not reach the maximum score of nine because of the rigid spine with restricted flexibility.

History analysis for all five probands showed early motor development delay, decrease of tendon reflexes, “floppiness”, and positive Gowers' sign. Myopathy did not progress with age. Intellectual development was normal in four children, and patient no. 3 (girl, 11 years) had a slight developmental delay – her IQ was 80 (normal values 85–115).

Apprehensive analysis of internal organs showed pathological alterations, the most notable were cardiovascular and bronchopulmonary problems. ECG detected sinus arrhythmia of varying degree in three patients (no. 1, 3, and 4). Echocardiography results revealed open *ductus arteriosus* in two patients (no. 1 and 2), and borderline (17mm) narrowed aorta at sinotubular junction (Patient no. 3). According to spirometry results, combined respiratory pulmonary function impairments were found in three probands. It was impossible to carry out spirometry for two patients due to their age (two and three years). Bilateral nephroptosis was detected in two children (Patient no. 1 and 2), left side nephroptosis was noted in one patient (no. 5) as well as midshaft hypospadia.

Hearing loss, another main feature of EDSKS2, was diagnosed in three patients (three, eleven, and fifteen years). Mild to moderate bilateral sensorineural hearing loss was revealed in the 11-year-old girl (Patient no. 3) and 15-year-old boy (Patient no. 4), mild bilateral mixed hearing loss was detected in a three-year-old boy (Patient no. 5). Ophthalmologic problems were diagnosed in three children, and the most severe alterations were in the 15-year-old boy (Patient no. 4). He had progressive high myopia with astigmatism and right side chorioretinitis without signs of inflammation. Patient no. 1 (three-year-old boy) had mild bilateral hypermetropia and Patient no. 5 (two-year-old boy) had mixed bilateral astigmatism.

Blood and urine tests, as well as biochemical analysis indicating basic metabolism levels, were normal in all five patients.

**Figure 3.**
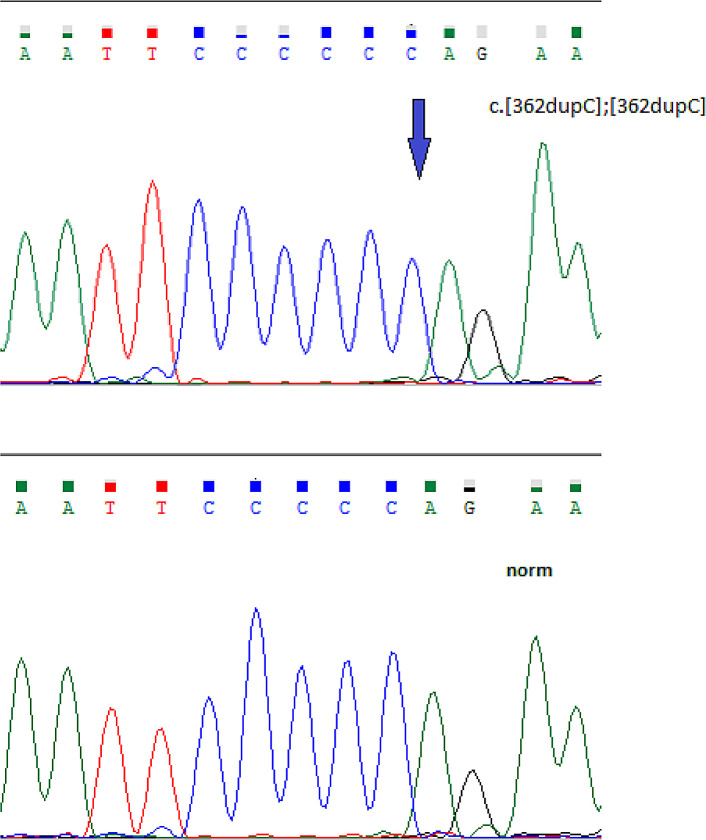
A homozygous c.362dupC pathogenic variant in exon 3 of the *FKBP14* gene.

A homozygous c.362dupC pathogenic variant in exon 3 of the *FKBP14* gene was detected in all five patients ( Accession number VCV000279809.13) ( Figure 3). ^6^

We present a brief highlight of a child’s clinical history (no. 4).

Z., a 15-year-old boy, was admitted to a clinic with complaints of progressive thoracolumbar kyphoscoliosis, *pectus excavatum*, muscular weakness, fatigue impairing his ability to engage in physical activities and to walk independently for a moderate period of time, vision and hearing impairments.

**Figure 4.**
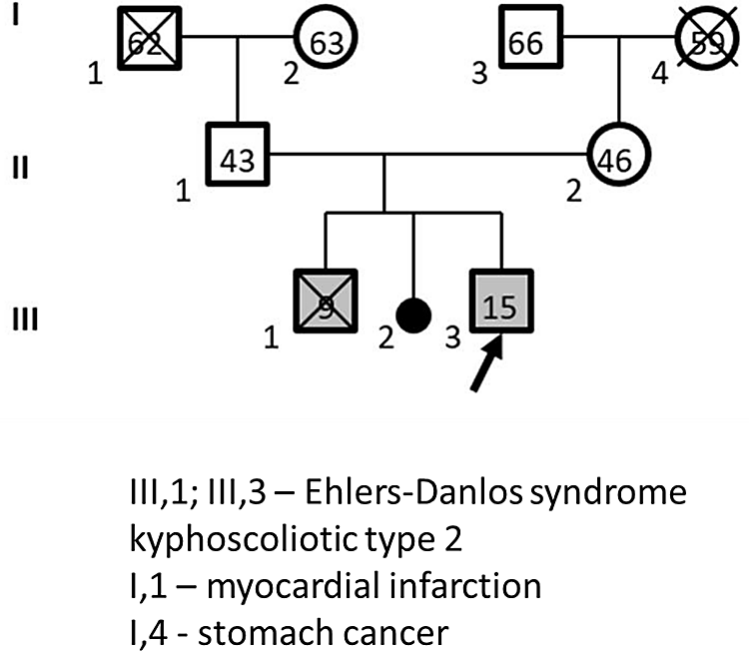
Pedigree of Proband no. 4, 15 years old. Roman numerals - generation number, Arabic numerals - family member number, numbers inside figures - family member's age, crossed out figures - deceased family member.

The proband was born to young healthy non-related parents ( Figure 4). His mother is currently 45-years-old, and his father is 41-years-old. The proband was from the third pregnancy, second delivery. It was established that the firstborn child was also male with the same clinical picture. He underwent multiple surgical operations to correct stage 4 kyphoscoliosis, but the last operation at the age of nine years was fatal.

The second pregnancy was aborted medically on the mother’s request. The third pregnancy was complicated by gestosis with risk of termination in trimesters I and II, but resulted in timely physiological delivery. The newborn’s mass was 3000 g, body length 51 cm, APGAR score 6/7. Neonatologists stated the child’s limbs were in the position of adduction. Early motor development was delayed: he started holding his head around the age of six months, sitting up at 12 months, walking independently at three years. At the age of two years progressive thoracolumbar kyphoscoliosis and *pectus excavatum* were noted. The child was under medical observation at the place of residence, received symptomatic therapy, including sanatorial treatment. However, the disorder kept progressing, and its origin was unclear to specialists. Some form of progressive muscular dystrophy was suggested; in order to obtain a clearer diagnosis, the patient was referred to the clinical genetics department of Veltischev Research and Clinical Institute for Pediatrics, Moscow, Russia.

**Figure 5.**
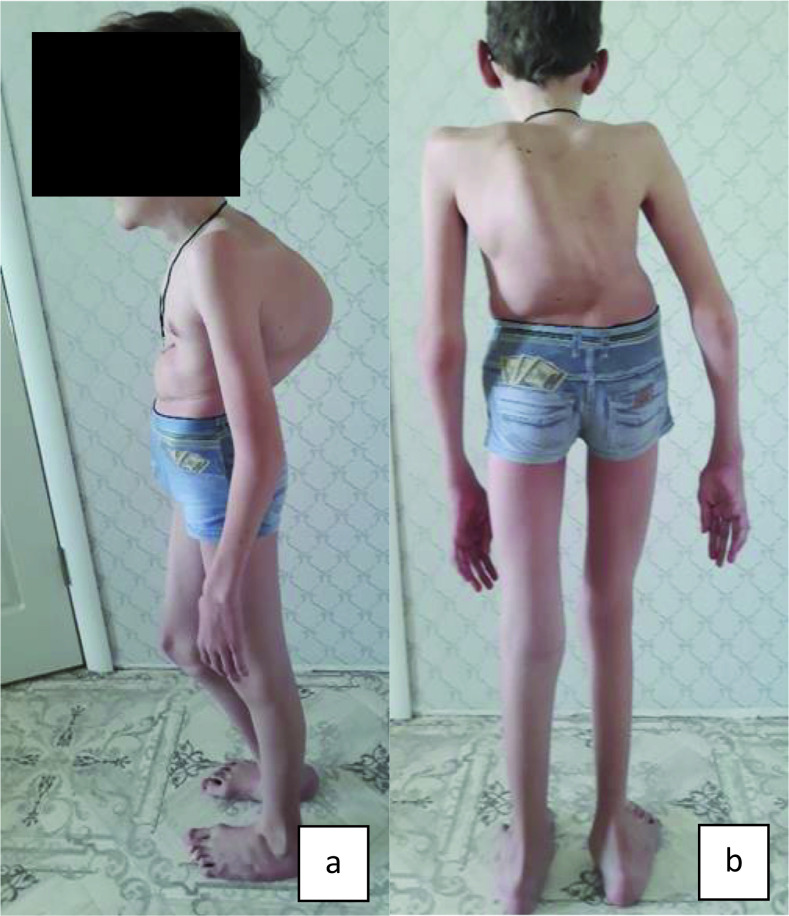
a) Proband no. 4: very low height and body mass (<3 percentile), thoracolumbar kyphoscoliosis of the 4 ^th^ degree, *pectus excavatum* of the 2 ^nd^ degree; b) Proband no. 4: thoracolumbar kyphoscoliosis of the 4 ^th^ degree, long thin limbs, *pes planovalgus*.

Upon admission, the proband’s condition was evaluated to be moderate to severe, in accordance with his main disorder. His physical development was very low, harmonic: body length - 140 cm, mass - 31 kg respectively (below third percentile). The following phenotypic features were the most notable ( Figure 5): thoracolumbar kyphoscoliosis of the 4th degree, *pectus excavatum* of the 2nd degree, *pes planovalgus*, joint hypermobility (eight on the Beighton scale), fatigue, muscular weakness resulting in Gowers’ maneuvers. Tendon reflexes were absent. Intellectual development corresponded to age.

Cardiac functional examination showed moderate sinus arrhythmia with periods of tachycardia, heart rate was 115–88 bpm. Echocardiography showed no heart defects: the chambers were not enlarged, the valves were intact, the contractory function of the myocardium was satisfactory, the diastolic function was normal, false chordae was detected in the left ventricle.

External ventilation function analysis showed profound combined respiratory pulmonary function impairment. Ultrasound examination of abdominal organs and kidneys did not reveal any pathology.

X-ray examination of the thoracolumbar spine region (dorsal-plantar and lateral) confirmed the presence of S-shaped stage 4 thoracolumbar scoliosis.

The electroneuromyography data indicated the muscular type of lesion: a slight decrease in the amplitudes of M-waves, a decrease in the amplitudes and duration of the motor unit action potentials (MUAPs), accelerated recruitment of the MUAPs; interference pattern analysis (IPA) indicators were below the standard limits.

Pure tone threshold audiometry showed mild to moderate bilateral sensorineural hearing loss. An ophtalmologist’s examination showed progressive high myopia with astigmatism, as well as OD chorioretinitis without signs of inflammation.

Blood and urine tests, as well as biochemical analysis indicating basic metabolism levels, were normal. Creatine kinase level was 167 upl, reference values 15–190 upl.

Clinical data suggested kyphoscoliotic type Ehlers-Danlos syndrome; it was also necessary to exclude congenital muscular dystrophy. The latter was suggested based on the congenital nature of the disorder, motor development delay, absence of tendon reflexes, muscular strength decrease, Gowers’ maneuvers, and electroneuromyography results. However, the slow muscular pathology progression with fast kyphoscoliosis progression (stage 4), normal CK levels, severe hypermobility syndrome (eight on the Beighton scale), and mild to moderate hearing loss called the diagnosis of primary muscular pathology into question. Molecular genetic analysis results showed a c.362dupC mutation in a homozygous state in exon 3 of the *FKBP14* gene. The boy’s parents had this mutation in a heterozygous state. These acquired results allowed us to confirm the EDSKS2 diagnosis.

The proband’s family was given medical genetic counselling, according to which the risk of an affected child was 25%. The couple was inclined towards preimplantation diagnostics.

## Discussion

In 2012, Baumann *et al*. ^5^ used genetic mapping to describe the *FKBP14* gene as a cause of Ehlers-Danlos syndrome kyphoscoliotic type. Since that moment, less than 30 such patients have been described in the medical literature. ^5, 7– 10^ To date, according to The Human Gene Mutation Database (HGMD), eight pathogenic variants are described in the *FKBP14* gene. The most common variant is the c.362dupC duplication, detected in 11 out of 17 patients by Giunta *et al*. (2018). ^9^ The majority of patients with this variant are Caucasian from various countries: UK, Austria, Croatia, Poland, etc. One Columbian patient has been reported. ^10^ The c.362dupC duplication was detected in a homozygous state in all five of our non-related patients from different regions of Russia. Baumann *et al*. (2012) ^5^ suggested the presence of a founder effect. However, a recurrent mutation caused by replication slippage in the polycytidine tract cannot be excluded.

The *FKBP14* gene encodes the FKBP22 protein, which is endoplasmic reticulum (ER) resident and controls post-translational modification of polypeptide chains containing hydroxyproline. FKBP22, a peptidyl-prolyl *cis-trans* isomerase, is a collagen folding catalyst and interacts with collagen types III, VI, and X. ^5, 11^ *FKBP14-*deficient skin fibroblast examination showed normal proportion and electrophoretic mobility of type I, III, and V collagen α-chains. Immunofluorescence revealed disorganisation of extracellular matrix proteins – collagen types I, III, VI, fibronectin, tenascins. Electronic microphotography of skin fibroblasts showed ER cisternae enlargement, fragmentation of elastic fibers. Symptoms of EDSKS1 and EDSKS2 are very similar: progressive kyphoscoliosis, severe congenital muscular hypotonia, hypermobility syndrome, skin hyperelasticity, medium arterial ruptures, osteopenia/osteoporosis. Diagnostic differentiation criteria: *PLOD1-*associated kyphoscoliotic type 1 - marfanoid phenotype, profound skin bruisability, scleral and ocular ruptures; *FKBP14*-associated kyphoscoliotic type 2 - hearing loss, follicular hyperkeratosis, muscular atrophy, bladder diverticulum. In addition, lysyl pyridinoline (LP) and hydroxylysyl pyridinoline (HP) excretion analysis may be useful - LP/HP ratio is increased in the case of kyphoscoliotic type 1.

Clinical findings in our patients corresponded to those described in the literature. ^2, 5^ The main symptom was progressive kyphoscoliosis in varying stages depending on the age. Three out of five patients had hearing loss, which is described in 73% of kyphoscoliotic type 2 cases. ^4^ The exact pathogenesis of this impairment is unclear. Three patients had osteoporosis; according to the literature, it is one of the minor diagnostic criteria, leading to fractures in 13% of cases. ^4, 9^ Three children had congenital malformations affecting three systems: cardiovascular (open *ductus arteriosus*, borderline narrowed aorta), skeleto-muscular ( *spina bifida sacralis dorsalis* S3-S5), and genital (midshaft hypospadia). Karyotype analysis and chromosomal microarray analysis did not show any abnormalities.

As shown in the literature, there are various types of Ehlers-Danlos syndrome with vascular complications (vascular, classic, classic-like, musculocontractural), which should be considered in differential diagnostics. ^1, 7, 12^ These complications in kyphoscoliotic type 2 patients could be caused by a pathogenetic link of the *FKBP14* gene with type III collagen, which is a significant component of vascular structure. ^13^

Kyphoscoliotic type 2 with symptoms of severe muscular atrophy has to be differentiated from Ullrich and Bethlem myopathy, which is caused by type VI collagen defects. The latter does not cause skin hyperelasticity, ecchymoses, hearing loss, but does cause striae, atrophic scars, respiratory abnormalities, and major joint contractures, uncharacteristic of EDSKS2.

Without any doubt, with such similarities in clinical pictures of EDSKS2 and the above-mentioned disorders, the final diagnosis has to be verified by molecular genetic analysis.

## Conclusion

Literature analysis and examination of five EDSKS2 patients demonstrated the involvement of major organs and systems, such as joints, spine, muscles, cardiovascular system, respiratory system, hearing, and vision, into the pathological process. Kidney mobility increases and nephroptosis seems to be secondary, caused by muscular weakness. During molecular genetic analysis, to verify EDSKS2 it is recommended to initially search for the c.362dupC duplication, which appears to be common in European countries, including Russia.

Many questions regarding the disorder's clinical polymorphism and progressive course remain unanswered. Some of them might be solved by a more detailed analysis of the *FKBP14* gene functions. The obtained information would improve our understanding of the disorder's pathogenetic mechanisms and aid in target therapy development.

## Data availability

### Underlying data

ClinVar: NM_017946.4(FKBP14):c.362dup (p.Glu122fs). Accession number VCV000279809.13; variation ID 279809; https://identifiers.org/clinvar:279809.

NCBI Nucleotide: Homo sapiens FKBP prolyl isomerase 14 (FKBP14), RefSeqGene (LRG_454) on chromosome 7. Accession number NG_032173.1; https://identifiers.org/ncbiprotein:NG_032173.1.

NCBI Nucleotide: Homo sapiens FKBP prolyl isomerase 14 (FKBP14), transcript variant 1, mRNA. Accession number NM_017946.4; https://identifiers.org/ncbiprotein:NM_017946.4.

4TU.ResearchData: Underlying data for: Ehlers-Danlos syndrome kyphoscoliotic type 2 caused by mutations in the *FKBP14* gene: an analysis of five cases. https://doi.org/10.4121/14705859.v1. ^6^

This project contains the following underlying data:

• Picture file 1. Gel of PCR fragments from patient no. 3. *FKBP14* gene, exons 1–4.
• Picture file 2. Electropherogram of the exon 3 of the *FKBP14* gene. A homozygous c.362dupC pathogenic variant in exon 3 of the *FKBP14* gene.
• Data file 1. Readme.pdf.

Data are available under the terms of the Creative Commons Zero “No rights reserved” data waiver ( CC0 1.0 Public domain dedication).

## Consent

### Consent for publication

A written informed consent for the publication of this manuscript including identifying images and other personal and clinical details was obtained from the participants and parents or legal guardians of all participants under the age of 18.
