# Supplementary material for: Ehlers-Danlos syndrome kyphoscoliotic type 2 caused by mutations in the FKBP14 gene: an analysis of five cases
Source: F1000Res. 2021 Jun 25;10:502. [Version 1] doi: 10.12688/f1000research.52268.1 (PMC8408539; doi:10.12688/f1000research.52268.1)
Supplement: Peer review history

# Peer review: Reviewer response for version 1

**DOI:** 10.5256/f1000research.55519.r90354

**Published:** 2021-08-31

**Authors:** Tomoki Kosho

**Affiliations:** 1 Department of Medical Genetics, Shinshu University School of Medicine, Nagano, Japan

The reviewer has evaluated a paper by Dr. Semyachkina, describing five patients with EDSKS2 caused by the same common variant in *FKBP14*. The disorder is very rare, and detailed clinical information would be valuable to understand the whole picture of the disorder including the natural history. Several issues have been found to be considered as follows:

1. X-ray images of the spine, hands, and feet had better be considered to be included.
2. How about the intellectual status of these patients, normal or subnormal?
3. How about craniofacial features of these patients, some similarities among the series?
4. How about skin involvement, hyperextensibility, fragility, and bruisability?

Is the work clearly and accurately presented and does it cite the current literature?

Yes

If applicable, is the statistical analysis and its interpretation appropriate?

Not applicable

Are all the source data underlying the results available to ensure full reproducibility?

Yes

Is the study design appropriate and is the work technically sound?

Yes

Are the conclusions drawn adequately supported by the results?

Yes

Are sufficient details of methods and analysis provided to allow replication by others?

Yes

Reviewer Expertise:

Clinical genetics

I confirm that I have read this submission and believe that I have an appropriate level of expertise to confirm that it is of an acceptable scientific standard.

# Peer review: Reviewer response for version 1

**DOI:** 10.5256/f1000research.55519.r88243

**Published:** 2021-07-12

**Authors:** Matthias Baumann

**Affiliations:** 1 Division of Pediatric Neurology, Department of Pediatrics I, Medical University of Innsbruck, Innsbruck, Austria

The manuscript presents in detail the findings in five additional patients with the rare kyphoscoliotic Ehlers-Danlos syndrome type 2 caused by mutations in the FKBP14 gene. They carried the most common c362dupC duplication. The patients of different age illustrate very well the spectrum of clinical symptoms. An interesting aspect, not well described so far, is the increased kidney mobility and nephroptosis.

 The paper helps to increase the awareness for this rare connective tissue disease, which is probably still underdiagnosed.

Is the work clearly and accurately presented and does it cite the current literature?

Yes

If applicable, is the statistical analysis and its interpretation appropriate?

Not applicable

Are all the source data underlying the results available to ensure full reproducibility?

Yes

Is the study design appropriate and is the work technically sound?

Yes

Are the conclusions drawn adequately supported by the results?

Yes

Are sufficient details of methods and analysis provided to allow replication by others?

Yes

Reviewer Expertise:

Neuromuscular disorders, Ehlers-Danlos-Syndrome

I confirm that I have read this submission and believe that I have an appropriate level of expertise to confirm that it is of an acceptable scientific standard.
